# The drug-transporter gene *MDR1* C3435T and G2677T/A polymorphisms and the risk of multidrug-resistant epilepsy in Turkish children

**DOI:** 10.1007/s11033-013-2866-y

**Published:** 2013-11-10

**Authors:** Mehmet Seven, Bahadir Batar, Selin Unal, Gozde Yesil, Adnan Yuksel, Mehmet Guven

**Affiliations:** 1 Department of Medical Genetics, Cerrahpasa Medical Faculty, Istanbul University, Istanbul, Turkey; 2 Department of Medical Biology, Cerrahpasa Medical Faculty, Istanbul University, Istanbul, Turkey

**Keywords:** Drug resistance, Epilepsy, MDR1, P-gp, Polymorphism

## Abstract

One-third of all individuals with epilepsy are resistant to antiepileptic drug (AED) treatment. Antiepileptic treatment response has been suggested to be modulated by genetic polymorphisms of drug efflux transporters. Several polymorphic variants within the multidrug resistance 1 (*MDR1*) gene, which encodes the major transmembrane efflux transporter P-glycoprotein, have been proposed to be associated with AED resistance in epilepsy patients. The aim of this study was to evaluate the effect of C3435T and G2677T/A polymorphisms of *MDR1* on AED resistance in Turkish children with epilepsy. *MDR1* C3435T and G2677T/A were genotyped in 152 patients with epilepsy, classified as drug-resistant in 69 and drug-responsive in 83. Genotypes of the C3435T and G2677T/A polymorphisms were determined by polymerase chain reaction followed by restriction fragment length polymorphism. Genotype and allele frequencies of C3435T and G2677T/A polymorphisms of the *MDR1* gene did not differ between drug-resistant and drug-responsive epilepsy patients. Our results suggest that *MDR1* C3435T and G2677T/A polymorphisms are not associated with AED resistance in Turkish epileptic patients. To clarify the exact clinical implication of the *MDR1* polymorphisms on the multidrug resistance in epilepsy, further investigations in various ethnic populations would be necessary.

## Introduction

Epilepsy is one of the most prevalent serious chronic neurological disorders, effecting approximately 1–2 % of the population [1]. About 30 % of patients with epilepsy do not respond to any of two to three first line antiepileptic drugs (AEDs) despite considerable advances in the therapy of epilepsy and these patients will develop refractory epilepsy [2]. Although efforts to predict pharmacoresistance have revealed several risk factors (an early onset of epilepsy, symptomatic or cryptogenic epilepsy, and generalized seizure) [3, 4], the mechanisms underlying the resistance to AEDs in the epilepsy treatment are still not well-understood.

Some potential mechanisms have been suggested regarding the transporters of the AEDs. There is accumulating evidence to suggest that an increase of functional expression of multidrug transporters produces pharmacokinetic changes that modify the access of AEDs to central nervous system targets [5]. Genetic polymorphisms in these transporters could account for their increased expression and/or functional activity. These genetic variations could be linked to changes on pharmacokinetic or pharmacodynamic of AEDs and effective responsiveness to AEDs [6].

The ATP-binding cassette (ABC) transporters, and especially ABCB1, are the focus of an international effort to establish their role in mediating drug resistance in a variety of human diseases including epilepsy [7]. Human multidrug resistant transporter gene (MDR1, also known as ABCB1), which is a member of the ABC superfamily, encodes a 170-kDa P-glycoprotein (P-gp) [8]. The P-gp is of particular interest, and was first identified in cancer cells, as a protein responsible for resistance to a wide variety of AEDs [9]. The transmembrane P-gp that functions as a multispesific drug-efflux transporter is considered to be associated with drug absortion and elimination. It is hypothesized that overexpression of an efflux transporter such as P-gp around an epiloptegenic focus, via reducing AED accumulation, may prevent AEDs from entering sites of action [10].

The expression, efflux, substrate spesificity, and mRNA stability of P-gp are influenced by various single nucleotide polymorphisms (SNPs) in the encoding gene, *MDR1* [11]. Evidence is present for supporting the important role played by more gene polymorphisms on the function of P-gp via effecting its activity [12, 13]. The *MDR1* gene is highly polymorphic with more than 50 variants residing in the coding region and, which can possibly cause altered function [14].

A great number of studies were focused on two most common SNPs, C3435T (Exon 26, rs1045642) and G2677T/A (Exon 21, rs2032582) SNPs, to explore the associations with disease development and drug response in different populations [10]. The synonymous C3435T polymorphism was the first SNP, which has been shown to correlate with the expression levels and function of P-gp [15], although conflicting reports have subsequently appeared showing no correlation with the expression levels and function of P-gp [12]. The C3435T polymorphism is in linkage disequilibrium with nonsynonymous G2677T/A polymorphism (Ala893Ser/Thr), which may also contribute to the observed functional effect of variation at the C3435T polymorphism or at associated haplotypes [16]. Furthermore, the G2677T/A SNP has been shown to be associated with drug resistant epilepsy [17, 18]. Thus, the *MDR1* has been regarded as an interesting candidate gene potentially influencing the response to AED treatment.

Based on the hypothesis that *MDR1* genetic polymorphisms can modulate drug response phenotype in epilepsy, the present study was carried out to evaluate the association of two SNPs [3435C > T (rs1045642) and 2677G > T/A (rs2032582)] of the *MDR1* gene in drug-responsive and drug-resistance patients in a Turkish population.

## Materials and methods

### Study population

A total of 152 Turkish children with epilepsy treated with AEDs were included in this study. The patients were diagnosed and classified according to the guidelines of the International League against Epilepsy. Patients, who had an established clinical diagnosis of epilepsy (defined by the occurrence of two or more unprovoked seizures) confirmed by an electroencephalogram examination by an expert were included in the study. Exclusion criteria consisted of severe adverse drug reactions, poor compliance with AEDs, history of pseudo-seizures, alcohol or drug abuse, or any other malignant diseases such as brain tumor, secondary metastasis, hepatic failure, or renal failure.

Patients were included if they had either drug-resistant or drug-responsive epilepsy according to the following definitions and criteria. The main criterion for drug resistance was the occurrence of at least four seizures over a period of 1 year with three AEDs, such as carbamazepine + valproate + levetiracetam, or carbamazepine + valproate + diphenylhydantoin, according to the epileptic seizure type at maximum tolerated doses. Of the 152 patients, 69 were classified as drug resistant (40 males, 29 females, mean age: 9.2 ± 3.6 years) and 83 were drug responsive (51 males, 32 females, mean age: 9.3 ± 3.8 years.

The protocol for this study was approved by the Regional Ethics Committee and was carried out in accordance with The Code of Ethics of the World Medical Association (Declaration of Helsinki) for experiments involving humans. An informed consent was signed by each participant or responsible adult.

### Blood samples and DNA isolation

Venous blood samples (3 ml) were collected in EDTA vacutainer tubes for DNA extraction. Immediately after collection, whole blood was stored in aliquots at −20 °C until use. Genomic DNA was extracted from leukocytes using Roche DNA purification kit (Roche Diagnostics GmbH, Mannheim, Germany) according to the manufacturer’s instructions.

### Genotyping

Genotyping of *MDR1* C3435T (exon 26) and G2677T/A (exon 21) were determined by using the polymerase chain reaction–restriction fragment length polymorphism (PCR–RFLP) assay. PCR amplifications of exon 26 and 21 of the *MDR1* gene were performed with primers described by Tanabe et al. and Cascorbi et al., respectively [19, 20]. PCR amplifications were carried out in a total volume of 25 μl containing: 100 ng of genomic DNA, 1U of *Taq* Polymerase (MBI Fermentas), 1 μM of each primer [for C3435T, F:5′-TTG ATG GCA AAG AAA TAA AGC-3′ and R:5′-CTT ACA TTA GGC AGT GAC TCG-3′; for G2677T/A, F:5′-TTT GCA GGC TAT AGG TTC CAG-3′, R(A):5′GTT TGA CTC ACC TTC CCA G-3′ and R(T):5′-TTT AGT TTG ACT CAC CTT CCC G-3′], 1X PCR buffer, 1 mM MgCl_2_, and 0.04 mM dNTPs. The amplification reactions were performed in a thermocycler 9700 (Applied Biosystems, California, USA) under the following conditions: For *MDR1* C3435T polymorphism, initial denaturation at 94 °C for 4 min, followed by 35 cycles each of 94 °C for 30 s, 56 °C for 30 s, 72 °C for 30 s; and a final extension at 72 °C for 5 min; for *MDR1* G2677T/A polymorphism, initial denaturation at 94 °C for 3 min, followed by 40 cycles each of 94 °C for 20 s, 55 °C for 20 s, 72 °C for 30 s; and a final extension at 72 °C for 5 min. The PCR products were digested by restriction endonucleases of *Mbo*I at 37 °C (for C3435T), *Ban*I at 37 °C (for G2677T) and *Bsr*I at 65 °C (for G2677A) for 3 h and analyzed with 3 % agarose gel electrophoresis.

### Statistical analysis

Mean and standard deviations (SDs) are presented in case of continuous variables. Differences between the means of the two continuous variables were evaluated by the Student’s *t* test. Chi square (χ^2^) or Fischer’s exact test (two-sided) was used to compare the association between the genotypes and alleles in relation to the cases, and test for deviation of genotype distribution from Hardy–Weinberg equilibrium. *P* values of <0.05 were considered statistically significant. The odds ratio (OR) and their 95 % confidence intervials (CIs) were calculated to estimate the strength of the association. Statistical analysis was performed using a statistical software package, SPSS 16.0 (SPSS Inc, USA). Haplotype frequency analysis and linkage disequilibrium for the two *MDR1* polymorphisms were performed by using the Haploview 4.2 program.

## Results

Basic demographic and clinical charecteristics of epilepsy patients with drug-resistant and drug-responsive are presented Table 1. The groups were not significantly different with respect to the age and gender (all values of *P* > 0.05).

**Table 1:** Demographic and clinical characteristics of epilepsy patients

Parameters	Drug-resistant *n* (%)	Drug-responsive *n* (%)
Gender		
Male	40 (58)	51 (61)
Female	29 (42)	32 (39)
Age (years)		
Mean ± SD	9.2 ± 3.6	9.3 ± 3.8
Range	1–15	2–14
Seizure type		
Partial	24 (35)	24 (29)
Generalized	45 (65)	59 (71)
Etiology		
Idiopathic	31 (45)	40 (48)
Symptomatic	29 (42)	27 (33)
Cryptogenic	9 (13)	16 (19)

*SD* standard deviation

No significant deviations from Hardy–Weinberg equilibrium were observed in epilepsy patients (*P* = 0.68 for *MDR1* C3435T and *P* = 1.00 for *MDR1* G2677T/A).

Table 2 shows the genotype and allele frequencies of the polymorphisms in *MDR1* gene for both drug-resistant and drug-responsive epilepsy patients. The distribution of *MDR1* C3435T and G2677T/A genotypes within drug-resistant (*P* = 0.76 and *P* = 1.00, respectively) and drug-responsive (*P* = 0.88 and *P* = 0.98, respectively) groups were not significantly different from that under Hary–Weinberg equilibrium. No statistically significant differences were detected for the genotype and allele frequencies of the polymorphisms in the *MDR1* gene between the drug-resistant and drug-responsive groups.

**Table 2:** Genotype and allele distribution of the MDR1 C3435T and G2677T/A polymorphisms in drug resistant and drug-responsive patients with epilepsy

Genotype/allele	Drug-resistant *n* (%)	Drug-responsive *n* (%)	OR (95 % CI)	*P*-value
C3435T				
CC	17 (25)	22 (26)	Ref.	
CT	30 (43)	38 (46)	1.02 (0.46–2.26)	0.96
TT	22 (32)	23 (28)	1.24 (0.52–2.93)	0.63
C allele frequency	0.46	0.49	Ref.	
T allele frequency	0.54	0.51	1.13 (0.65–1.97)	0.67
G26677T/A				
GG	17 (25)	20 (24)	Ref.	
GT	32 (46)	36 (43)	1.05 (0.47–2.33)	0.91
TT	17 (25)	24 (29)	0.83 (0.34–2.04)	0.69
GA	1 (1)	2 (3)	0.59 (0.05–7.07)	1.00
TA	2 (3)	1 (1)	2.35 (0.20–28.27)	0.60
AA	0 (0)	0 (0)	–	–
G allele frequency	0.49	0.47	Ref.	
T allele frequency	0.49	0.52	0.90 (0.52–1.58)	0.72
A allele frequency	0.02	0.01	1.92 (0.17–21.87)	1.00

*OR* odds ratio, *CI* confidence interval

Table 3 lists the haplotypes of *MDR1* polymorphisms in the drug-resistant and drug-responsive epilepsy patients. Haplotype frequencies of these polymorphisms in the *MDR1* gene did not differ significantly between drug-resistant and drug-responsive epilepsy patients. The linkage disequilibrium test of two SNPs in *MDR1* (Rs1045642 and rs2032582) were in strong linkage disequilibrium (D′ = 0.991).

**Table 3:** MDR1 C3435T and G2677T/A haplotype frequencies in drug-resistant and drug-responsive patients with epilepsy

Haplotype	Frequency		*P*-value
	Drug-resistant	Drug-responsive	
TT	0.535	0.526	0.988
CG	0.409	0.404	0.972
TG	0.044	0.035	0.487
CT	0.012	0.035	0.235

## Discussion

Drug-resistant epilepsy patients tend to be resistant to a broad range of AEDs with a varied mode of action [5, 7]. The functional characteristics of non-specific drug transporter proteins could explain broader resistance across types of epilepsy and to a range of AEDs [21]. Among these transporter proteins, P-gp is considered as one of the most potent contributer to the phenomenon of clinical drug resistance to AEDs.

Various genetic polymorphisms in the *MDR1* gene could be linked with altered in vivo transport activity of P-gp which may play a facilitatory role in drug-resistant epilepsy [6]. These genetic polymorphisms among epilepsy patients are likely to contribute to different responses in patients, who have similar epilepsy syndromes and are taking similar, or even the same, doses of medication.

So in the present study, we investigated two SNPs in *MDR1* gene in drug-resistant and drug-responsive epilepsy patients. Results of the present study demonstrated that genotype and allele frequencies of C3435T and G2677T/A polymorphisms of the *MDR1* gene did not differ between drug-responsive and drug resistant epilepsy patients. Furthermore, the results of this study also showed no haplotype association of these *MDR1* polymorphisms with drug-resistant epilepsy.

The results from various studies focusing on the role of *MDR1* C3435T and G2677T/A polymorphisms in AED responsiveness are conflicting. Consistent with the present study, several studies have found no significant associations between drug-resistance epilepsy and the *MDR1* C3435T and G2677T/A polymorphisms [22, 23]. Two studies from Turkey also reported that there were no associations between the polymorphisms of the *MDR1* gene and drug-resistant epilepsy [24, 25]. Similarly, Kim et al. [26] observed no association between the polymorphisms of G2677T/A and 3435C >T and drug resistance in Korean patients. However, contrary to the above studies’ results, Hung et al. [17] found that 3435C >T and 2677G >T in the *MDR1* gene contributed to drug-resistant epilepsy. Siddiqui et al. [27] demonstrated that the frequency of the 3435CC genotype among drug-resistant patients (27.5 %) was significantly higher than in drug-responsive patients or nonepileptic controls (15.7 or 18.5 %, respectively) among Caucasian subjects. In contrast to these results, Kwan et al. [28] reported that patients with drug-resistant epilepsy had a higher frequency of the 3435TT genotype (16.7 %) in comparison to drug-responsive epileptics or nonepileptic controls (10.6 or 7.4 %, respectively). Furthermore, Seo et al. also demonstrated that drug-resistant epilepsy patients were significantly more likely to have the T allele and the TT genotype at C3435T when compared to drug-responsive patients (50 vs 27; 36.9 vs 16.7 %, respectively). In addition, Seo et al. [29] found that patients with drug-resistant epilepsy had higher frequencies of the TT genotypes at G2677T/A in comparison to the drug-responsive patients (26.2, 23.8 %, respectively).

The differences observed in these reports may be due to the ethnic differences in genotype/allele frequencies. Genetic polymorphisms often vary between ethnic groups. Ethnic differences in genotype frequency for the investigated polymorphisms might effect the results in genetic studies. Distribution of *MDR1* C3435T polymorphism also displays an ethnic difference. For example, *MDR1* 3435CC was less common than 3435TT in Caucasian patients (16–19 vs 27–33 % in drug-responsive patients) [22, 27], while the opposite of this was observed in Han Chinese cohort (38 vs 7 % in drug responsive patients) [28], similar to other Asian populations [30]. Furthermore, contradictory data reported in these epidemiologic studies may be due to other several factors such as number of patients, combinations of susceptibility variants, the isolated nature of the study populations, heterogeneity of epilepsy and gene-environment interactions.

The authors assessed the association of *MDR1* haplotypes derived from C3435T, G2677T/A, and C1236T loci in response to AEDs in different epilepsy populations [31]. In consistency with our results, Alpman et al. [25] found that the haplotypes of C3435T and G2677T/A were not associated with drug resistant epilepsy in Turkish epileptics. Furthermore, Meng et al. [32] failed to detect any associations between drug-resistance and haplotypes of C3435T, G2677T/A, and C1236T polymorphisms. However, Siddiqui et al. [27] reported that the C–G–C haplotype of C3435T, G2677T/A, and C1236T was associated with the AED-resistance. Zimprich et al. [18] also identified that C–G–C haplotype significantly increased the risk for pharmacoresistance. Contrary to this, Seo et al. [29] found that patients with drug-resistant epilepsy had higher frequencies of the T–T–T haplotype at C3435T, G2677T/A, and C1236T than patients with drug-responsive epilepsy (42.1, 30.4 %, respectively).

In conclusion, our results indicate that *MDR1* C3435T and G2677T/A polymorphisms are not associated with drug-resistant epilepsy in the study population. The limitation of the current study is the small sample size. To clarify the exact clinical implication of the MDR1 polymorphisms on the multidrug resistance in epilepsy, further investigations in various ethnic populations would be necessary.
